# Zoonotic onchocerciasis in Hiroshima, Japan, and molecular analysis of a paraffin section of the agent for a reliable identification

**DOI:** 10.1051/parasite/2011182185

**Published:** 2011-05-15

**Authors:** M. Fukuda, Y. Otsuka, S. Uni, T. Boda, H. Daisaku, H. Hasegawa, H. Takaoka, O. Bain

**Affiliations:** 1 Research Promotion Project, Oita University Hasama, Yufu Oita 879-5593 Japan; 2 Department of Infectious Disease Control, Faculty of Medicine, Oita University Hasama, Yufu Oita 879-5593 Japan; 3 Department of Medical Zoology, Osaka City University Medical School Abeno-ku Osaka 545-8585 Japan; 4 Shobara Red Cross Hospital Shobara Hiroshima 727-0013 Japan; 5 Department of Biology, Faculty of Medicine, Oita University Hasama, Yufu Oita 879-5593 Japan; 6 Institute of Biological Sciences, Faculty of Science, University of Malaya Kuala Lumpur 50603 Malaysia; 7 Parasitologie comparée et Modèles expérimentaux, USM307, Muséum National d’Histoire Naturelle 75231 Paris France

**Keywords:** zoonotic onchocerciasis, *Onchocerca dewittei japonica*, mitochondrial DNA analysis, Japan, onchocercose zoonotique, *Onchocerca dewittei japonica*, analyse de l’ADN mitochondrial, Japon

## Abstract

Japan is a country of high specific diversity of *Onchocerca* with eight species, the adults of two not yet known. *Onchocerca dewittei japonica*, a common filarial parasite of wild boar, had been proved to be the agent of five zoonotic onchocerciasis in Kyushu island with morphological and molecular studies. The sixth case, at Hiroshima in the main island, was identified to the same *Onchocerca* species, based on adult characters observed on histological sections. To consolidate the identification, mitochondrial cytochrome *c* oxidase subunit 1 (CO1) gene analysis was attempted with the formalin-fixed, paraffin-embedded parasite specimen. The sequence (196 bp) of a CO1 gene fragment of the parasite successfully PCR-amplified agreed well with those of *O. dewittei japonica* registered in GenBank, confirming the morphological identification. Moreover a comparison with the CO1 gene sequences of six other *Onchocerca* species in GenBank excluded the possibility that *Onchocerca* sp. from wild boar and *Onchocerca* sp. type A from cattle in Japan, were the causative agents in this case. Mitochondrial DNA analysis proved to be a valuable tool to support the morphological method for the discrimination of zoonotic *Onchocerca* species in a histological specimen.

## Abstract (fr)

Le Japon est une région de grande diversité du genre *Onchocerca* avec huit espèces, dont deux identifiées par les microfilaires, les adultes étant encore inconnus. Il a été démontré par les analyses morphologiques et moléculaires qu’*Onchocerca dewittei japonica*, filaire fréquente du cochon sauvage, est l’agent des cinq cas d’onchocercose zoonotique dans l’île de Kyushu. Le sixième cas, à Hiroshima, île principale, a été identifié à la même espèce d’onchocerque d’après les caractères de l’adulte observés sur coupes histologiques. Afin de vérifier cette identification, l’analyse du gène mitochondrial de la sous-unité 1 de la cytochrome *c* oxydase (CO1) a été effectuée avec une coupe du specimen fixé au formol et inclus en paraffine. La séquence (196 bp) d’un fragment du gène CO1 du parasite, amplifié par PCR, est semblable à celles d’*O. dewittei japonica* enregistrées dans GenBank, confirmant l’identification morphologique. En outre, la comparaison avec les séquences du gène CO1 de six autres espèces d’*Onchocerca* déposées dans cette base de données exclut comme agents possibles *Onchocerca* sp. du sanglier et *Onchocerca* sp. type A du bétail au Japon. L’analyse de l’ADN mitochondrial est donc un outil valable qui complète la méthode morphologique de discrimination des espèces zoonotiques d’onchocerques sur coupes histologiques.

Human zoonotic onchocerciasis is rare. Only 16 cases have so far been reported in the world, including six cases in Japan, five from Oita, Kyushu, and the most recent from Hiroshima (Uni *et al.*, 2010). The causative agents of all the Japanese cases were identified as *Onchocerca dewittei japonica* Uni, Bain & Takaoka, 2001, a common filarial parasite of wild boar (*Sus scrofa* Linnaeus) in Japan (Uni *et al.*, 2001), based on the morphological characteristics of the adult worms (Beaver *et al.*, 1989; Hashimoto *et al.*, 1990; Takaoka *et al.*, 1996, 2001, 2004, 2005; Uni *et al.*, 2010). As for the first two cases, the species was confirmed retrospectively because at that time *O. dewittei japonica* had not yet been discovered (Takaoka *et al.*, 2001; Uni *et al.*, 2010).

In Japan, six other *Onchocerca* species are known (Takaoka *et al.*, 2005; Uni *et al.*, 2007): three cosmopolitan parasites of domestic animals, *O. cervicalis* Railliet and Henry, 1910 from horses, *O. gutturosa* Neumann, 1910, and *O. lienalis* (Stiles, 1982) from cattle; three of wild animals, *O. eberhardi* Uni & Bain, 2007 from sika deer (*Cervus nippon* Temminck), *O. skrjabini* Rukhlyadev, 1964 from sika deer and serows (*Capricornis crispus* Temminck), and *O. suzukii* Yagi, Bain & Shoho, 1994 from serows.

Recently another *Onchocerca* species was found from wild boars in Japan (Fukuda *et al.*, 2008, 2010a). This unnamed species, the adult of which is unknown, is distinguishable from *O. dewittei japonica* by the body size of the microfilaria (Fukuda *et al.*, 2008). In addition, there is another unnamed *Onchocerca* species (its adults unknown) found from cattle in Japan (Takaoka & Bain, 1990). This, designated as type A, is also distinguished from other *Onchocerca* species by the morphology of the microfilaria and the infective larva (Takaoka & Bain, 1990; Fukuda *et al.*, 2010b). Thus, there remains the possibility that either of these two unnamed species was involved as the causative agent of all or some of six Japanese cases so far reported. On the other hand, we have already shown that both of these two unnamed species are distinguishable from *O. dewittei japonica* by the mitochondrial cytochrome *c* oxidase subunit 1 (CO1) gene analysis (Fukuda *et al.*, 2010a, 2010b).

In order to investigate the possibility of one of the two unnamed *Onchocerca* species being the causative agent of the sixth case of zoonotic onchocerciasis in Japan (Uni *et al.*, 2010), we performed the mitochondrial DNA analysis for a formalin-fixed, paraffinembedded parasite specimen.

## Materials and Methods

### Specimen examined

**Fig 1.**
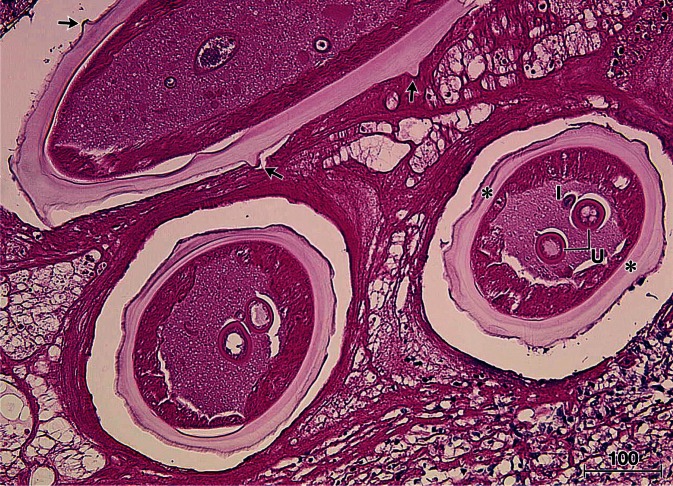
Histological section of a nodule showing the worm and surrounding tissue excised from the patient. Morphological characteristics of the female worm identified as *Onchocerca dewittei japonica* showing the salient transverse ridges (arrows), lateral chords (*), uteri (U), and intestine (I). The section is stained with hematoxylin and eosin. Bar, micrometers.

A tissue sample stored as a paraffin block of the sixth case of zoonotic onchocerciasis in Japan was examined, where the worm found in the histological sections had been already identified as female *O. dewittei japonica* based on its morphology (Fig. 1) (Uni *et al.*, 2010). In short, in July 2009, a subcutaneous nodule (2 cm in diameter) was surgically removed from the left knee of the patient, a 70-year-old man living in Hiroshima Prefecture, Japan. The tissue excised (1 × 2 cm) was fixed in 4% paraformaldehyde for 24 hr and embedded in paraffin by a routine procedure (Uni *et al.*, 2010). For molecular analysis the section was cut at thickness of 20 μm.

### DNA extraction

The tissue of the worm (ca. 2.8 mm2) was scraped from the section on a glass slide with a disposable sterilized scalpel blade and transferred into a 1.5 ml microcentrifuge tube. The tissue was incubated with 0.5 ml of DEXPAT (Takara Bio Inc., Otsu, Japan) for 10 min at 100 °C and then centrifuged for 10 min at 12,000 rpm at 4 °C. Ten microliters of the supernatant was used as template DNA for PCR.

### PCR and Sequencing of the Partial Mitochondrial CO1 Gene Region

Two primer sets, general filarial primers CO1intFCO1intR (Casiraghi *et al.*, 2001) and newly designed CO1fF (5’-TTGTCTGTTCCTGTTTTGG-3’)-CO1fR (5’- GCAAAAGTTATTCTAGTTTGACCA-3’) respectively, were used to amplify a fragment of the mitochondrial CO1 gene (coding sequence). CO1fF-CO1fR was constructed inside CO1intF-CO1intR on the basis of the known sequences of *Onchocerca* species in Japan. The positions of the primers on the complete mitochondrial genome of *O. volvulus* (GenBank accession number: AF015193) are: CO1intF, 2519-2538; CO1intR, 3207-3186; CO1fF, 2884-2902; CO1fR, 3099-3122. Amplifications were performed in 50 μl containing 1 × buffer for KOD -Plus- Ver.2 (Toyobo, Osaka, Japan), 1.5 mM MgSO4, 200 μM each of dNTPs, 0.1 μM each of primers, 0.5 units of KOD -Plus- (Toyobo), and 10 μl of template DNA. The thermal conditions were as follows: larger fragments (689 bp), an initial denaturation at 94 °C for 2 min, followed by five cycles of 98 °C for 10 s, 55 °C for 30 s, and 68 °C for 45 s and 37 cycles of 98 °C for 10 s, 48 °C for 30 s, and 68 °C for 45 s; smaller fragments (239 bp), an initial denaturation at 94 °C for 2 min, followed by five cycles of 98 °C for 10 s, 60 °C for 30 s, and 68 °C for 30 s and 37 cycles of 98 °C for 10 s, 55 °C for 30 s, and 68 °C for 30 s.

PCR products were purified with a QIAquick PCR Purification Kit (QIAGEN, Hilden, Germany) and directly sequenced using the primers for PCR, a BigDye Terminator v3.1 Cycle Sequencing Kit (Applied Biosystems, Foster City, CA, USA), and an Applied Biosystems 3130 Genetic Analyzer (Applied Biosystems). PCRs were conducted twice and each of the amplification products were sequenced. The sequence determined was deposited in DDBJ/EMBL/GenBank databases under the accession number AB604943 (Table 1).

**Table 1.** Nucleotide differences over 196 sites of the CO1 gene sequences among *Onchocerca* species in Japan.

		1	2	3	4	5	6	7	8	9	10
**1**	This study (*O. dewittei japonica* Hiroshima*, AB604943)		0	1	2	19	21	15	18	20	20
**2**	*O. dewittei japonica* (AB518874)	0		1	2	19	21	15	18	20	20
**3**	*O. dewittei japonica* (AM749266)	0.5	0.5		1	18	20	14	17	19	19
**4**	*O. dewittei japonica* (AB518875)	1.0	1.0	0.5		17	21	13	18	18	18
**5**	*O*. sp. wild boar	9.7	9.7	9.2	8.7		20	13	18	13	18
**6**	*O*. sp. type A	10.7	10.7	10.2	10.7	10.2		20	15	15	14
**7**	*O. eberhardi*	7.7	7.7	7.1	6.6	6.6	10.2		17	12	13
**8**	*O. suzukii*	9.2	9.2	8.7	9.2	9.2	7.7	8.7		15	11
**9**	*O. skrjabini*	10.2	10.2	9.7	9.2	6.6	7.7	6.1	7.7		8
**10**	*O. gutturosa*	10.2	10.2	9.7	9.2	9.2	7.1	6.6	5.6	4.1	

Values above the diagonal are the numbers of nucleotide differences, and those below the diagonal are the percentages of nucleotide differences.

* Diagnosis by morphological observation.

### Data Analysis

The sequence obtained was aligned with published sequences of seven *Onchocerca* species in Japan. Using this alignment, sequences were compared by MEGA 4.0.2 based on 196 bp available for comparison (Tamura *et al.*, 2007). Used GenBank database accession numbers were as follows: *O. dewittei japonica* (AM749266, AB518874, AB518875), *O. eberhardi* (AM749268), *O. gutturosa* (AJ271617), *O. skrjabini* (AM749269), *O.* sp. type A *sensu* Fukuda *et al.*, 2010 (AB518876), *O.* sp. wild boar *sensu* Fukuda *et al.*, 2010 (AB518693), and *O. suzukii* (AM749275).

## Results and Discussion

The mitochondrial CO1 gene was not amplified with the CO1intF-CO1intR primers (expected size: 689 bp), which are proved to generate products from various filarial species (Casiraghi *et al.*, 2001; Fukuda *et al.*, 2010a, 2010b). This was probably due to the degradation of DNA because the specimen (Fig. 1) was fixed in paraformaldehyde and embedded in paraffin (Bianchi *et al.*, 1991). Thus the new primers, CO1fF-CO1fR, were designed inside the CO1intF-CO1intR and a 239 bp-gene fragment was successfully amplified. The sequence (excluding primers) determined was 196 bp long and was compared with those of all seven *Onchocerca* species of Japan available in GenBank. Table 1 shows the nucleotide differences among *Onchocerca* species in Japan. The numbers of nucleotide differences between the present specimen and *O. dewittei japonica* ranged from 0 to 2, small enough to assure the conspecific status, while those between the present specimen and other *Onchocerca* species ranged from 15 to 21. The differences between the present specimen and the two undescribed species, *O.* sp. from wild boar and *O.* sp. type A from cattle, were 19 and 21, respectively, suggesting that neither of them was the causative species.

The causative agents of zoonotic onchocerciasis are relatively difficult to identify morphologically, particularly if the available specimens are restricted to histological sections, or the fauna of *Onchocerca* species and their definitive natural hosts in the areas where zoonotic onchocerciases occurred are unknown, as shown in most of the previous cases of zoonotic onchocerciasis. On the other hand, molecular analysis can directly identify the causative species as shown by Koehsler *et al.* (2007) who identified *O. jakutensis* (Gubanov, 1964), a parasite of red deer (*Cervus elaphus* Linnaeus), as the causative species from a histological section of a patient in Austria.

In the present study, we could also prove that DNA analysis of mitochondrial CO1 gene region was a very effective method for the accurate identification of the causative species of zoonotic onchocerciasis in an area where the relevant molecular data are comparatively well documented on *Onchocerca* species including ones of which adults remain unknown. Although we used a 20 μm-thick section of paraffin-embedded tissue for molecular analysis to investigate the possibility of the two undescribed species as a causative agent, species identification from a section with a usual thickness of 4 μm may be possible, but is yet to be confirmed. This type of molecular analysis will be a useful tool for the definitive diagnosis in similar cases of zoonotic onchocerciasis in future.

In conclusion, our study suggests that mitochondrial DNA analysis is a useful tool to support the traditional morphological method for species identification of the causative agent of zoonotic onchocerciasis in view of the fact that few specimens, especially histological sections, are available and the life cycle of the causative species is uninvestigated.
